# Process optimization and enhancement of pesticide adsorption by porous adsorbents by regression analysis and parametric modelling

**DOI:** 10.1038/s41598-021-91178-3

**Published:** 2021-06-03

**Authors:** Mohammad Hadi Dehghani, Amir Hessam Hassani, Rama Rao Karri, Bahareh Younesi, Mansoureh Shayeghi, Mehdi Salari, Ahmad Zarei, Mahmood Yousefi, Zoha Heidarinejad

**Affiliations:** 1 Department of Environmental Health Engineering, School of Public Health, Tehran University of Medical Sciences, Tehran, Iran; 2 Center for Solid Waste Research, Institute for Environmental Research, Tehran University of Medical Sciences, Tehran, Iran; 3 Department of Environmental Engineering, Faculty of Environment and Energy, Science and Research Branch, Islamic Azad University, Tehran, Iran; 4 Petroleum and Chemical Engineering, Faculty of Engineering, Universiti Teknologi Brunei, Bandar Seri Begawan, Brunei Darussalam; 5 Department of Medical Entomology and Vector Control, School of Public Health, Tehran University of Medical Sciences, Tehran, Iran; 6 Student Research Committee, Department of Environmental Health Engineering, School of Public Health, Hamadan University of Medical Sciences, Hamadan, Iran; 7 Department of Environmental Health Engineering, School of Health, Social Determinants of Health Research Center, Gonabad University of Medical Sciences, Gonabad, Iran; 8 Food Health Research Center, Hormozgan University of Medical Sciences, Bandar Abbas, Iran; 9 Department of Environmental Health Engineering, Faculty of Health, Hormozgan University of Medical Sciences, Bandar Abbas, Iran

**Keywords:** Climate sciences, Environmental sciences

## Abstract

In the present study, the adsorptive removal of organophosphate diazinon pesticide using porous pumice adsorbent was experimentally investigated in a batch system, modelled and optimized upon response surface methodology (RSM) and artificial neural network-genetic algorithm (ANN-GA), fitted to isotherm, kinetic and thermodynamic models. The quantification of adsorbent elements was determined using EDX. XRD analysis was utilized to study the crystalline properties of adsorbent. The FT-IR spectra were taken from adsorbent before and after adsorption to study the presence and changes in functional groups. The constituted composition of the adsorbent was determined by XRF. Also, the ionic strength and adsorbent reusability were explored. The influences of operational parameters like pH, initial pesticide concentration, adsorbent dosage and contact time were investigated systematically. ANN-GA and RSM techniques were used to identify the optimal process variables that result in the highest removal. Based on the RSM approach, the optimization conditions for maximum removal efficiency is obtained at pH = 3, adsorbent dosage = 4 g/L, contact time = 30 min, and initial pesticide concentration = 6.2 mg/L. To accurately identify the parameters of nonlinear isotherm and kinetic models, a hybrid evolutionary differential evolution optimization (DEO) is applied. Results indicated that the equilibrium adsorption data were best fitted with Langmuir and Temkin isotherms and kinetic data were well described by pseudo-first and second-order kinetic models. The thermodynamic parameters such as entropy, enthalpy and Gibbs energy were evaluated to study the effect of temperature on pesticide adsorption.

## Introduction

The enormous growth in population was putting the world under stress, to meet their global food demand. To cater this demand, food production has to be increased many folds, and crop losses due to pests should be reduced. Around the globe every year more than 800 different agricultural pesticides are produced. Among them, organophosphate pesticides are extensively used due to their significant effect on a wide range of pests, high durability, and low cost^1^. Unfortunately, the excessive use of these pesticides will accumulate in the soil and subsequently end up in water bodies during the rainfall-runoff, thus polluting water and the environment. Chlorpyrifos, malathion, chlorpyriphos-methyl, and diazinon are among the essential organophosphate pesticides that are commonly found in the surface and underground water resources^2^. Organophosphate pesticides pose a severe threat to human health due to central nervous system dysfunction and cholinesterase activity. According to global statistics, the highest mortality due to pesticides is associated with organophosphate pesticides. Diazinon pesticide is one of the extensively used organophosphate pesticides for agricultural purposes around the world^1,3,4^. Typically, the half-life of the insecticide in natural water is around 180 days at pH = 7.4. However, diazinon quickly hydrolyze in the aquatic environment within 12 h under acidic environment (pH = 3.1). Diazinon is non-polar, slightly water-soluble, and resistant to decomposition in soil. A lethal dose of diazinon for humans has been reported 90–444 mg/kg of body weight. Thus, the concerns on surface and groundwater pollution and ultimately endangering the human health and aquatic creatures are a global issue, which force the global researchers to further explore their mitigation^3^.

So far, various methods have been investigated for removing recalcitrant organic pollutants from an aqueous environment, including photocatalytic processes^5,6^, oxidation^7^, electrochemical decomposition^8^, membrane separation^9^, and adsorption^10,11^. Some of these methods are complex processes, which incur a high cost as well as higher consumption of chemicals. In this regard, researchers have found that the adsorption process is a more efficient method, and its operating costs can be reduced by using various natural as well as synthetic adsorbents^12^.

Among the various natural and low-cost adsorbents, Pumice was considered as a natural adsorbent for adsorption process as it is a lightweight material (density ~ 0.5—1 kg​​/L) with a high porosity (φ = 85%)^13^. It is structurally composed of a series of irregular cavities, some of them connected to each other, and volcanic gases like sulfur-based are being confined in them. Pumice stone has a porous structure and large surface to volume ratio that make it a potential candidate as a natural and low-cost adsorbent^14^. Till today, many studies have been conducted on the performance of pumice for the removal of water pollutants^13,15,16^, but the removal of organophosphate diazinon pesticides using pumice has not been reported so far in the open literature.

Therefore, the prime objective of this research study is to evaluate the performance of pumice as an adsorbent for organophosphorus diazinon pesticide removal from water bodies. In this study, the influence of independent process variables (initial diazinon concentration, pH, contact time, and adsorbent dosage) on the overall efficiency is investigated systematically. To capture the intrinsic features of the pumice adsorption process and minimize the experiments, response surface methodology (RSM) is used. To further predict the natural pumice removal at different operating conditions and identify the optimal process variable values, backpropagation artificial neural network (ANN) is used as a data-driven model. Also, the interaction effect of process variables is thoroughly investigated, and these variables are optimized using RSM and artificial neural network-genetic algorithm (ANN-GA). To understand the mechanism involved in pesticide adsorption by pumice, isotherms and kinetic modelling are examined separately. To accurately identify the parameters of nonlinear isotherm and kinetic models, a hybrid evolutionary differential evolution optimization (DEO) is applied. To minimize the use of pumice, regeneration studies are conducted to validate its performance in removing diazinon for multiple cycles. Also, an ionic strength study was conducted to simulate the real condition of aquatic environments during the adsorption process.

## Experimental section

### Materials and chemicals

The laboratory-grade chemicals like NaOH, H_2_SO_4_, HNO_3_ used in this study are purchased from Merck Company (Germany), and diazinon pesticide (60% purity) purchased from Partonar Company, Iran. Natural Pumice (Fig. S1) was obtained from Tekmeh Dash, East of Azerbaijan. The constituted components of pumice used in this study were found to be SiO_2_ (71.75%), Al_2_O_3_ (12.33%), Fe_2_O_3_ (1.98%), CaO (0.7%), MgO (0.12%), Na_2_O (3.59%), K_2_O (4.47%), and SO_3_ (0.18%).

### Adsorbent preparation

Initially, solid pumice was crushed and sieved through a mesh size of 10–70 (200 to 2000 μm) and was rinsed with distilled water several times to remove unwanted impurities. In order to increase the porosity of the adsorbent, pumice was placed in 1 N H_2_SO_4_ at room temperature for two days. Then, it was washed thoroughly until the effluent turbidity is reduced to be clear and can read under spectrometer, as well as pH reaches neutral conditions. Then, the adsorbent was dried in an oven for about 8 h at 105 °C^17^.

### Adsorbent characterization

The porosity and density of pumice were measured according to the following procedure. The saturation and Buoyancy technique was employed for pumice porosity and density, which conforms to the standard suggested by ISRM and ASTM^18^. The mass of pumice sample was 50 g and irregular in form. The pumice bulk volume (V) was determined by measurement of the saturated‐submerged mass (Msub) and the saturated mass (Msat) of the pumice sample. Initially, the pumice sample is immersed in water to be saturated under vacuum conditions of less than 800 Pa for 1 h. Regular mixing was done to remove trapped air. The mass of saturated‐submerged pumice was measured to determine Msub. The pumice sample is taken out of the water and the mass of the pumice plus container (B) is measured with an accuracy of 0.01 g. The pumice sample is then dried in the open container located in an oven for 24 h at the temperature of 105 °C to reach the constant mass. The container, with the dry pumice, was cooled in a desiccator for 30 min, and the mass (C) of the dry sample with the container is again measured with an accuracy of 0.01 g. Also, the mass of the container (A) was determined separately. The following calculations were done to obtain the porosity and dry density of the pumice sample.

1 $$ Msat = B - A $$

2 $$ Ms = C - A $$

3 $$ V = \frac{Msat - Msub}{{\rho_{w} }} $$

4 $$ V_{V} = \frac{Msat - Ms}{{\rho_{w} }} $$

5 $$ \rho_{d} = \frac{Ms}{V} $$

6 $$ n = \frac{{V_{V} }}{V} \times 100 $$

Here, V and V_V_ represent the total volume and pore volume, respectively, of the pumice sample (cm^3^). ρ_d_ and n_e_ stand for dry density (g/ cm^3^) and porosity percent, respectively. Ms, Msub, and Msat show the dry, saturated‐submerged, and saturated masses, respectively, of the pumice sample (g). A, B, and C are the mass of the container, dried pumice sample plus container, and saturated pumice sample plus container, respectively, (g). Geometries, morphology, and structural characteristics of pumice adsorbent were analyzed using field electron emission microscopy (SU-3500 model, HITACHI). The quantification of adsorbent elements was determined using an Energy dispersive X-ray spectroscopy device (EDX, Ametext, Oktane prime model). X-ray diffraction (XRD) analysis (Rigaku miniflex diffractometer device) was utilized to study the crystalline properties of pumice. The FT-IR spectra were taken from pumice before and after adsorption by using Perkin-Elmer Spectrophotometer to investigate the presence and changes in functional groups. The constituted composition of the pumice was determined by a Philips X-ray fluorescence (XRF) analysis (Philips PW1480 model). pH at the point of zero charge (pH_ZPC_) was determined by the pH drift method for pumice adsorbent. 180 mL of 0.01 M NaCl solution was added in 650 mL beakers, and the pH of solutions was adjusted by NaOH/H_2_SO_4_ to be within the range of 2 to 12. Then, 0.25 g of the adsorbent was added to each Erlenmeyer and was mixed for 24 h at 120 rpm, and the final pH is recorded.

### Batch experiments

All batch experiments on diazinon adsorption by pumice adsorbent were performed in Erlenmeyer flasks with a volume of 250 mL. The stock solution of 50 mg/L was prepared by liquifying 0.025 g of diazinon in 500 mL deionized water. The process parameters like initial diazinon concentration, pH, contact time, and pumice dosage were explored in the adsorption process at the levels given in Table S1 and the experiment design given in Table S2. The sample was filtered with a 0.45 μm syringe filter, and the remaining diazinon in the solution was measured using DR 5000™ UV–Vis spectrophotometer at 248 nm wavelengths^12^. Then, the efficiency of diazinon removal and adsorption capacity by pumice adsorbent was determined. For every experimental run according to the experiment design (Table S2), one sample was conducted under the designed experiment conditions without adding the pumice. Its UV absorption was measured at 248 nm after filtration using 0.45-micron filter paper. By comparing the UV absorption between the samples and the standard curve, no reasonable change in the UV absorption was identified, indicating that there was no self-settling of pesticide due to its limited solubility. Moreover, the designed experiments were repeated by adding the pumice to the samples without having the pesticide to identify whether UV absorption at 248 nm occurs by the added pumice. After shaking and filtering the samples, it confirmed that there was no background resulted from pumice powder at 248 nm.

### Experimental design

In the current study, central composite design (CCD) in RSM was employed for designing the experiments. Applying this method via a statistical approach optimizes the number of required experimental runs and hence reduces the wastage of chemicals and lowers the total budget^19^. Furthermore, this method identifies the relationship between the process variables and removal efficiency, as well as identifies the interactive effects within the variables and overall influence on the removal efficiency. The process variables that influence the removal efficiency were identified as initial diazinon concentration, contact time, pH, and pumice dosage. Therefore, these process variables were considered within the ranges, which were categorized as five levels (Table S1), and thus, results in an experimental matrix of 30 experimental runs (Table S2) with different values of process variables. Each experiment was performed thrice to minimize the measurement/experimental error and to check the variation among the outcomes. It was observed that the variability of these outcomes is less than 2%; hence the average value was used for further analysis studies and reported in Table S2.

### Implementation of a hybrid ANN-GA algorithm

The multivariate modeling of the experimental design was performed by the ANN modeling, which can establish a data-driven model which can capture the interdependency of process parameters and their effect on the response^20–22^. Here, a three-layered (hidden, input, and output layers) ANN model was implemented with the Levenberg–Marquardt backpropagation algorithm with 1000 epochs. Tansig and Purelin transfer functions were employed at the hidden and output layer, respectively^23^. Among the total data, a set of 70%, 15% and 15% data were randomly segregated for training, validation, and testing, respectively. The number of neurons in the hidden layer remarkably influences the model structure and hence the predicted values. Therefore, the best number of neurons (1 ~ 14) in the hidden layer was verified via well-known statistical metrics called mean squared error (MSE) and coefficient of determination (R^2^). The best optimum number of neurons will result in a lower value of MSE and the higher value of R^2^ (close to 1.0).

The performance of ANN depends on the appropriate choice of the weights and bias in the ANN architecture. Instead of the manual trial & error method, these weights can be more estimated (optimized) by suitable optimization techniques. In this study, a genetic algorithm is used to optimize the weights, which can find the exact or estimated relation between the response (removal efficiency) and input process variables. In this method, computing solutions are based on the population and get updated at every iteration if it is better than the former one. This procedure is continued until the best solution or fit is achieved. GA utilizes individual populations obtaining from different combinations of process variables, thus explores all search space for the best solution. This configuration is denoted as a hybrid ANN-GA technique^24–26^.

### Isotherms, kinetics, and Thermodynamics studies

The study of adsorption isotherms can better understand the mechanisms involved in the diazinon adsorption onto pumice. Hence, the adsorption equilibrium data were fitted with six well-known adsorption equilibrium isotherms. The model expressions of these isotherms are presented in Table S3. Also, four kinetics models (Table S4) are evaluated and fitted in order to understand the inherent kinetics further and interpret the rate of adsorption of solute onto the adsorbent surface.

Temperature being an essential factor in the adsorption process, so to understand its effect on the performance of natural pumice adsorbent for diazinon removal, the experimental studies are conducted at 20, 30, 40, and 50 °C. The thermodynamic parameters such as entropy change (ΔS°), enthalpy change (ΔH°) and Gibbs energy change (ΔG°), were evaluated to study the effect of temperature on the diazinon adsorption^27,28^. The expression to calculate the parameters was given in supplementary material.

### Evaluation of isotherms and kinetic models

Usually, most researchers evaluate the isotherm and kinetic model parameters by linearizing the non-linear equation. However, linearization will misinterpret the adsorption mechanisms as well as the adsorption rate. So, to use the same non-linear model expression, the model parameters are evaluated by well-known differential evolution optimization (DEO). The features of DEO and its implementation are provided in ESM as well as from previous studies^29^. The performance of DEO based model predictions are validated with important statistical metrics, as shown below:

7 $$ Coefficient\,of\,correlation\,{(}R^{2} ) = \frac{{\sum\nolimits_{i = 1}^{n} {\left( {q_{e,pred}^{i} - q_{e,exp}^{i} } \right)^{2} } }}{{\sum\nolimits_{i = 1}^{n} {\left[ {\left( {q_{e,pred}^{i} - \overline{q}_{e,\exp }^{i} } \right)^{2} } \right]} }} $$

8 $$ Root\,mean\,square\,error\,(RMSE) = \sqrt {\frac{1}{n - 1}\sum\limits_{i = 1}^{n} {\left( {q_{e,pred}^{i} - q_{e,\exp }^{i} } \right)^{2} } } $$

9 $$ Sum\,of\,absolute\,error\,(SAE) = \sum\limits_{i = 1}^{n} {\left| {q_{e,pred}^{i} - q_{e,\exp }^{i} } \right|} $$

10 $$ Sum\,of\,absolute\,error\,(SSE) = \sum\limits_{i = 1}^{n} {\left( {q_{e,pred}^{i} - q_{e,\exp }^{i} } \right)}^{2} $$

11 $$ Average\,relative\,error\,(ARE) = \frac{100}{p}\sum\limits_{i = 1}^{n} {\frac{{\left| {q_{e,pred}^{i} - q_{e,\exp }^{i} } \right|}}{{q_{e,exp}^{i} }}} $$

where *n* and *p* is the number of experimental runs and parameters; $q_{e,exp}^{i} ,\overline{q}_{e,exp}^{i} ,q_{e,pred}^{i}$ are the q_e_ values of the experimental, average and predicted values, respectively”;

## Results and discussion

### Adsorbent characterization

**Figure 1:**
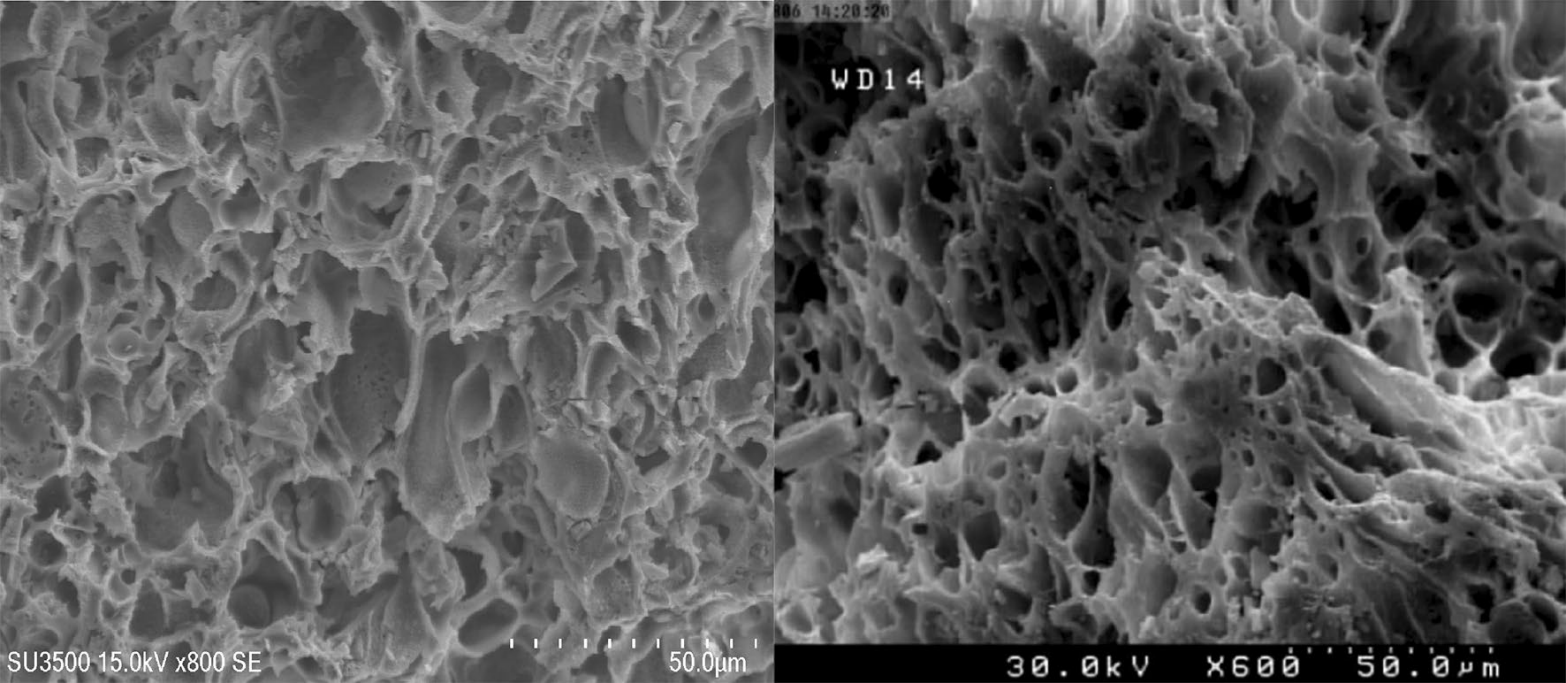
SEM graphs of pumice adsorbent.

The porosity and density of pumice were measured to be in the range of 50–85% and 0.7–1.2 g/cm^3^ according to the saturation and Buoyancy technique. SEM image (Fig. 1) of pumice adsorbent showed a porous surface with large grains and sharp edges on the adsorbent.

**Figure 2:**
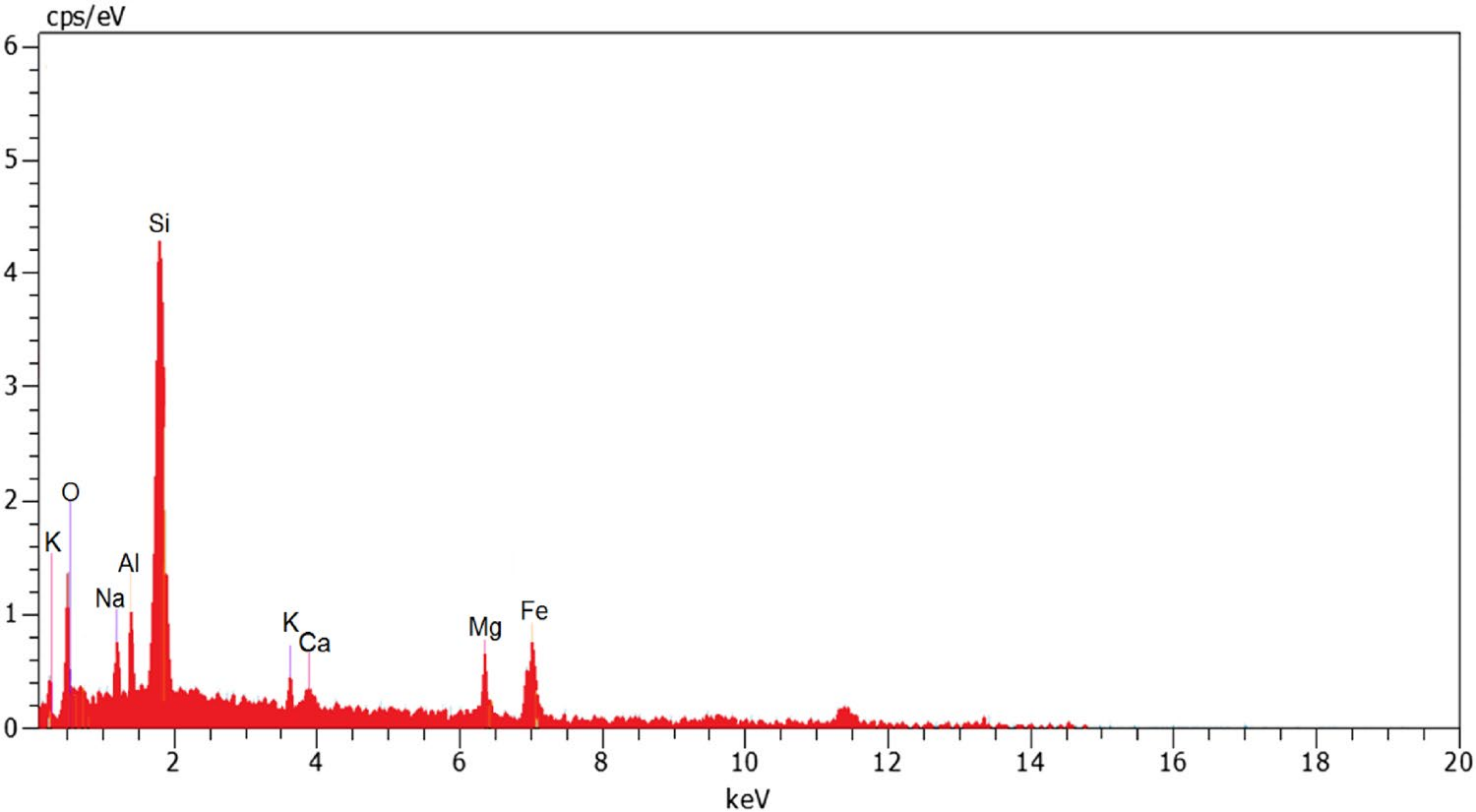
EDX analysis of pumice.

**Figure 3:**
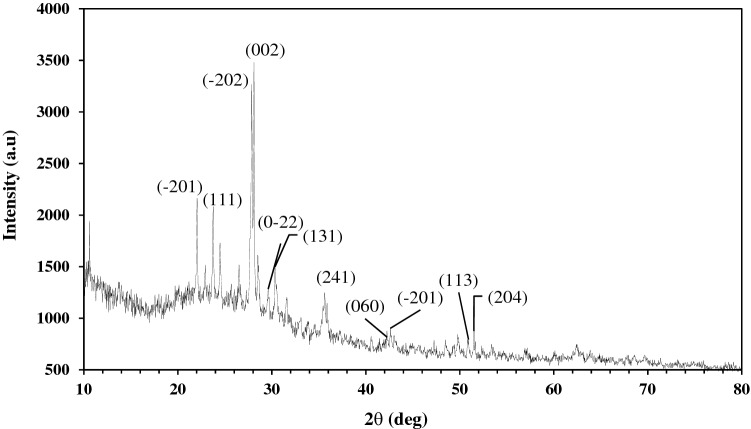
The XRD pattern of natural pumice.

**Figure 4:**
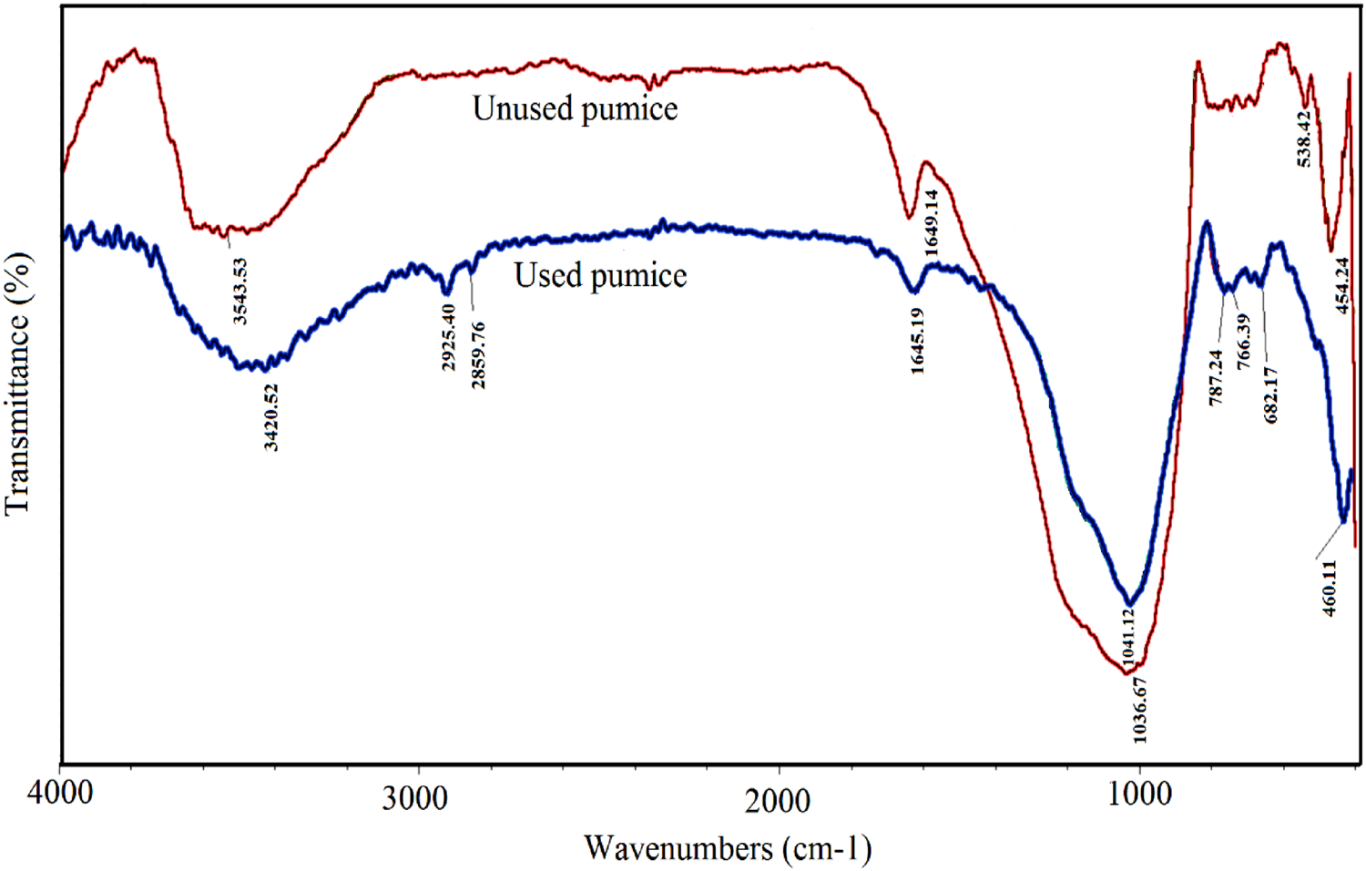
FTIR spectrum for used and unused pumice.

The results of the EDX analysis shown in Fig. 2 elucidated that the elemental composition of pumice are O (45.08%), Si (34.47%), Al (7.13%), K (3.17%), Mg (2.59%), Fe (2.55%), Na (2.53%) and Ca (1.48%). XRD pattern for pumice was depicted in Fig. 3. The results show that some diffraction peaks at the crystalline pattern of the pumice have appeared at 2θ of 22.1, 23.7, 27.8, 28.1, 30.1, 30.4, 35.8, 50.3, and 52.3, which belong to (100), (002), (101), (102), (110), (103), (200), (112) and (201) crystalline planes and correspond to the reference XRD pattern with the standard JCPDS code of 00–041-1481. Accordingly, the main component of pumice was found to be anorthite (Na.25Ca.71(Al2Si2O8)), a kind of natural zeolite constituted from sodium, calcium, aluminum, and silicate^30^. In addition, the background line has formed a broad peak in the 20°–30° range, points out amorph substance. Figure 4 depicts FTIR spectra of pumice before and after adsorption. The bands that appeared at 3543.53 cm^-1^ are ascribed to the stretching vibration of the H–O group. The peaks around 1649.14 cm^-1^ can also be allocated to the stretching vibration of H–O groups arising from water absorbed^31^. The peaks that appeared at 1035–1045 cm-1 bonds are assigned to stretching vibrations of Si–O and Al-O, and between 400 and 500 cm-1 bonds are related to their bending modes. The Si–O-Al stretching vibration is found around 787–788 cm -1 bonds^32^. By making a comparison between the FTIR spectra of used and unused pumice, the peaks at 3543.53 cm-1 and 1649.14 cm-1 bonds, which are related to the OH group, although could be observed, were found to be weaker and shifted to 3420.52 cm-1 and 1645.19 cm-1 bonds, respectively. The results reveal that electrostatic interactions play a key role in the adsorption of diazinon in acidic pHs owing to the existence of positively charged pumice^33^. It also observed that two new peaks had been appeared at 2925.40 cm^-1^ and 2859.76 cm^-1^ bands of FTIR spectrum of used pumice, corresponding to asymmetric and symmetric stretching vibrations of C–H bonds, respectively. This confirms the residues of adsorbed diazinon onto the pumice surface^34^.

### Model fitting and statistical analysis

Based on the RSM-CCD framework, a quadratic model (Eq. 12) is developed that shows the connection between the output response and process variables. Based on the analysis of variance (ANOVA) approach, the following quadratic expression is obtained.

12 $$ {\text{Diazinon}}\,{\text{removal}}\,{\text{efficiency}} = 32.577 - 4.588{\text{X}}_{1} + 1.547{\text{X}}_{2} + 8.249{\text{X}}_{3} + 0.272{\text{X}}_{4} - 0.055{\text{X}}_{1} {\text{X}}_{2} + 0.256{\text{X}}_{1} {\text{X}}_{3} {-}0.005{\text{X}}_{1} {\text{X}}_{4} + 0.043{\text{X}}_{2} {\text{X}}_{3} {-}0.005{\text{X}}_{2} {\text{X}}_{4} + 0.015{\text{X}}_{3} {\text{X}}_{4} + 0.158{\text{X}}_{1}^{2} {-}0.013{\text{X}}_{2}^{2} {-}1.011{\text{X}}_{3}^{2} {-} \, 0.024{\text{X}}_{4}^{2} $$

The Pareto effect of each process variable (factor) on the removal efficiency was obtained according to Eq. (13), which is shown in Fig. S2.

13 $$ {\text{P}}_{{\text{i}}} = \left[ {\frac{{({\text{b}}_{{\text{i}}}^{2} )}}{{\sum {\text{b}}_{{\text{i}}}^{2} }}} \right] \times 100 $$

where *b* shows the regression coefficient of each term. As displayed in Fig. S2, the initial diazinon concentration has the highest effect (about 51.8%) on the removal efficiency.

The statistical results of the analysis of variance (Table S5) indicate a well-fitted model, and most of the factors in the model have a substantial effect on the outcome. The R^2^ value for this model was 0.9997, which concludes the high capability of the model in predicting the response variability. The residuals are situated near/on the 45° line, confirming the normality of error (Fig. S3a). Also, the residuals vs. predicted (Fig. S3b) plot signifies that the residuals are erratically scattered around the baseline and do not follow any specified trend.

### Data-driven modelling by ANN

To configure optimal ANN topology, the different number of neurons (1–14) in the hidden layer is initially verified. Simulation runs indicate that the 8-neuron hidden layer model results in the lowest mean square error. Therefore, these eight neurons are chosen as an optimum number of neurons in this ANN topology. Hence, a three-layered feed-forward network with a 4:8:1 topology (Fig. S4) was employed for modeling the process. The performance of the ANN model is displayed in Fig. S5. It can be observed that most of the predicted values are close to 45° line, which thus resulted in R^2^ of 0.999, 0.995, and 0.976 for training, validation, and testing, respectively. The model predictions are in good compatibility with the experimental values and also better than RSM model predictions, thus indicating the higher capability of capturing the non-linear dynamics of the adsorption system.

### Performance of model predictions by RSM and ANN approaches

Based on the quadratic model (Eq. 8), the pesticide removal efficiency by pumice adsorbent is estimated, and the performance of model predictions against the experimental values is shown in Fig. S6a. These RSM predictions are in good fit with the experimental values, resulting in an R^2^ of 0.986. Similarly, the three-layered 4:8:1 topology ANN model predictions were compared against the experimental values, as shown in Fig. S6b. It can be noticed that these predictions are as good as experimental values, hence resulted in a higher R^2^ of 0.992. To evaluate the performance of both RSM and ANN, their residual errors (model predictions – experimental values) were calculated and plotted for each experimental run, as shown in Fig. S6c. These residual errors further confirm that the superior performance of the data-driven model derived from ANN.

### Effect of pH_ZPC_ for pumice adsorbent

The final pH value of the solution is compared to the initial pH to determine pH_ZPC_ for pumice adsorbent (Fig. S7). In the figure, the point where the final pH value is equal to the initial pH is considered as pH_ZPC_. In this research, the obtained value of pH_ZPC_ for pumice adsorbent was equal to 6. When the pH of the solution < pH_ZPC_, the adsorbent surface charge is positive and vice versa^35^. The surface of pumice is +ve charged at a solution pH values below 6 (pH_ZPC_) and would, therefore, be prone to the electrostatic attraction of diazinon molecules^10^. At pH over 6, the surface charge would be negative. These negative charges on the sites decrease the adsorption of anionic diazinon because of electrostatic repulsion. This finding is similar to those reported by other studies^13,36^. Therefore, at the solution, pHs are less than pH_ZPC_ (3 <6) and more than pKa of diazinon, the adsorbent surface has a positive charge.

In contrast, diazinon molecules begin to decompose into anionic species and are adsorbed by electrostatic adsorption on active sites of pumice adsorbent^36,37^. Therefore, the maximum removal of diazinon occurs under acidic pH conditions, and these outcomes in this study are consistent with the results of other studies^38^. The mechanism for adsorption of diazinon on pumice can also be in the interlayer adsorption space of pumice as well as its surface. This is consistent with the results of previous works^39^.

### Interaction effect of process variables

**Figure 5:**
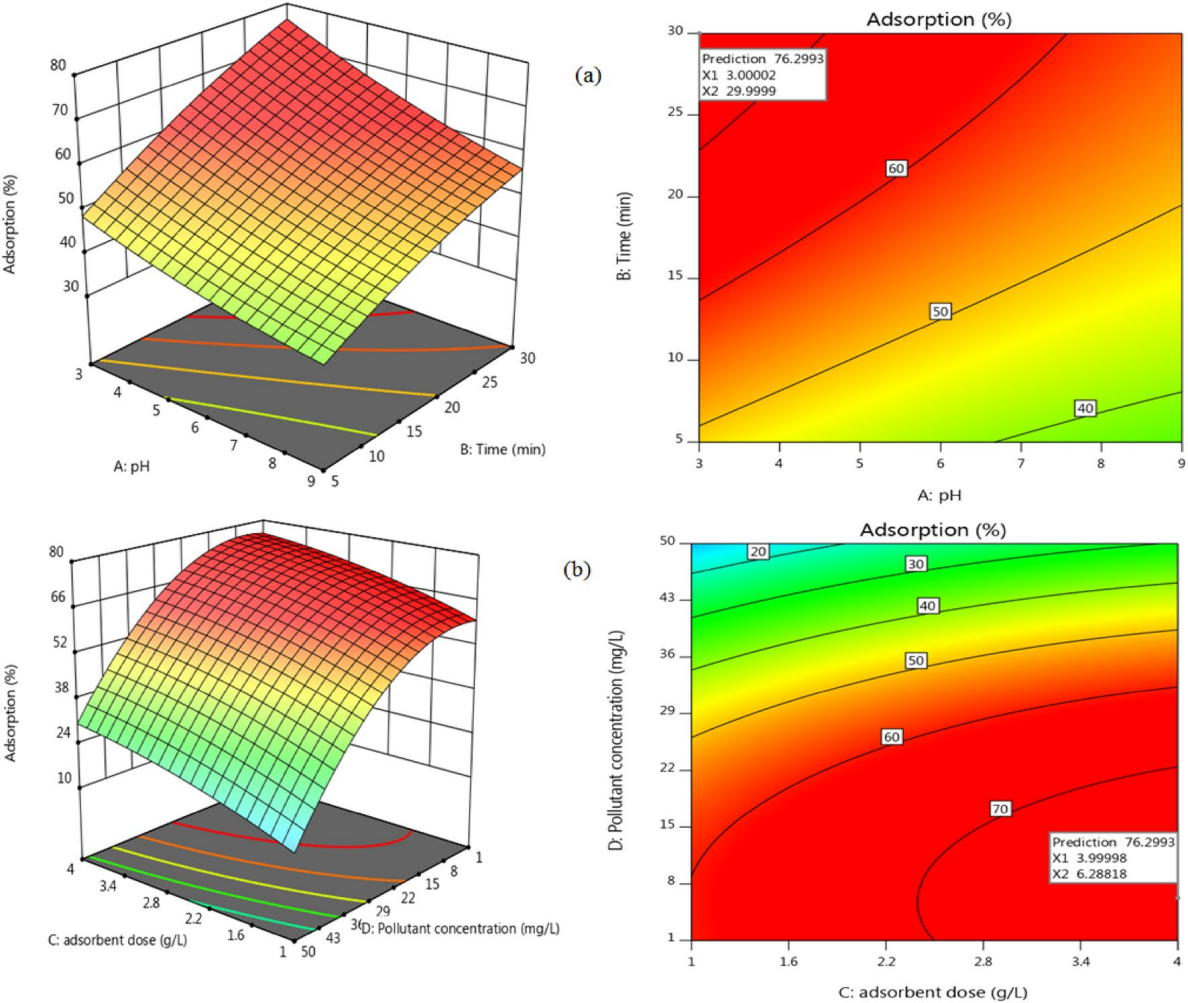
The 3D response surface and contour plots for diazinon removal by pumice, (**a**) pH -contact time on adsorption (initial diazinon concentration = 6.28 mg/L, and pumice dosage = 4 g/L), (**b**) pumice dosage—initial diazinon concentration on adsorption (pH = 3 and contact time = 30 min).

To better visualize the interaction effect of process variables on the overall diazinon removal efficiency, the contour plots and 3D surface plots are produced as given in Fig. 5. As seen in Fig. 5a, the maximum removal efficiency of diazinon was observed in acidic conditions, and by increasing pH, the removal of diazinon exhibits a reducing trend. This behaviour can be attributed to various reasons: 1) rapid hydrolysis of diazinon under acidic conditions and 2) a pKa of 2.6 of diazinon^40^.

Contact time is one of the essential variables in the uptake of diazinon pesticide by pumice adsorbent. As seen in Fig. 5a, the amount of diazinon removal increased rapidly at the early stages and then gradually reached a steady state. With increasing contact time from the start of the process to longer times, diazinon removal increased. This fact could be due to the presence of empty adsorption sites on the adsorbent during initial contact time and the increased rate of transfer of diazinon molecules from the solution to the adsorbent surface. It must be noted that the removal rate at a prolonged time is relatively lower than the initial times because the sites on the adsorbent are saturated at the extended contact time, and fewer sites are available to adsorb the pollutant^41^. These results are consistent with the results reported in other studies on the diazinon removal by other adsorbents, as well as the use of pumice to remove other pollutants than diazinon^16^.

The amount of diazinon adsorption at different initial concentrations of diazinon is shown in Fig. 5b. According to the trends shown in this figure, it is observed that with increasing the concentration of diazinon from 1 to 7 mg/L, diazinon removal by pumice has increased, then with increasing the concentration, the removal efficiency of diazinon gradually reduced. This can be due to the fact that with increasing the concentration of diazinon, competition has been increased to adsorb diazinon in the adsorbent active sites, and adsorption capacity increased, but adsorption reduced due to increased diazinon molecules in the environment^42^. This result is consistent with the trend reported in another study^16^.

The adsorbent dosage is also one of the key variables in determining the quantity of removal of a selected pollutant^41,43^. Figure 5b shows the amount of Diazinon adsorption as a function of pumice adsorption dosage. According to the trends seen in this figure, it is found that diazinon removal increased with increasing the pumice dosage. This increase can be attributed to enhanced active sites while increasing the adsorbent dosage; hence the amount of diazinon adsorption increased. Also, the continuous reduction in adsorption capacity with increasing the dosage may be due to the lower access adsorbate molecules to the adsorbent surface in higher doses^44^.

### Optimization studies by RSM-GA approaches

Keeping the maximization of removal efficiency as a desirable goal, the process variables were optimized. Using the RSM-CCD approach, along with the quadratic model, the optimal values of process variables are evaluated. As observed in Fig. S8, the max diazinon removal efficiency is 76.3% which can be attained at the optimal values of 6.28 mg/L, 4 g/L, 3, and 30 min for initial diazinon concentration, pumice dosage, pH and contact time, respectively.

Optimization by the genetic algorithm was carried out for each parameter within the range given in Table S6. The optimization can result in maximum pesticide adsorption. Figure S8 depicts the significant statistical factors in the implementation of the GA technique. By implementing GA, the optimized values that are obtained for initial diazinon concentration, pumice dosage, pH, and contact time are 6.266 mg/L, 4 g/L, 3, and 30 min, respectively. At these conditions, the maximum pesticide removal was obtained as 76.5%. These findings were consistent with the optimization values that resulted in the RSM technique. To validate the consistency of the optimal predicted responses by both RSM and GA approaches, three experiments were conducted at these optimum conditions (as per RSM), and the average adsorption efficiency was found to be 77.0% (± 1.7) (Table S6). These results are close to the predicted responses of RSM and GA approaches. Moreover, at the optimum operational parameters and neutral pH values in the range of 6 to 8, removal efficiency changed from 64.9 to 59.3%.

### Effect of ionic strength

**Figure 6:**
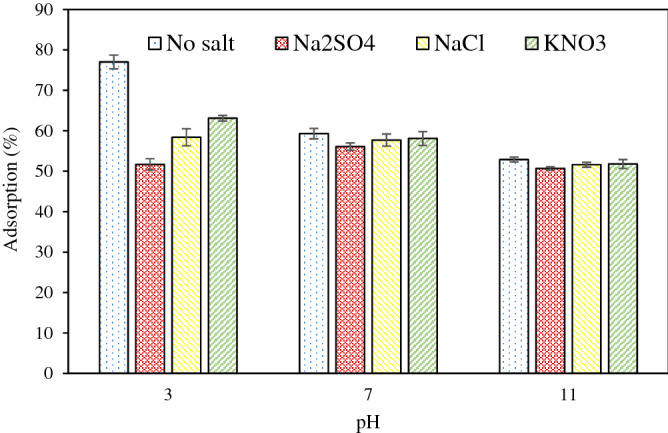
Effect of inorganic salts on diazinon adsorption on pumice (initial diazinon concentration = 6.28 mg/L, pumice dosage = 4 g/L, and contact time = 30 min, electrolyte salt concentration = 500 mg/L).

The existence of various salts in the effluent might affect the adsorption efficiency. Since various salts exist in aqueous solutions, one of the important propose of the present study was to examine the adsorption efficiency of diazinon onto the pumice in the presence of the different electrolytes including NaCl, Na_2_SO_4,_ and KNO_3_ at acidic, neutral and basic pHs. The results (Fig. 6) elucidate that the removal efficiency is suppressed significantly at acidic pH, whereas it exhibits no significant decrease at neutral and basic pHs. The considerable reduction in removal efficiency at acidic pH indicates that electrostatic attraction potentially contributes to the diazinon adsorption onto the pumice. The electrostatic interaction indeed occurs between the adsorbed H^+^ groups and the anionic form of diazinon molecules at acidic pH^45^. Theoretically, when the electrostatic attraction prevails between adsorbent and adsorbate, the added salts compete with adsorbate for the occupation of the vacant sites on the adsorbents, thus decreasing the overall adsorption efficiency^46^. The results also confirm that the adsorption at the system at acidic pH is of outer-sphere surface complexation. As stated, no significant decrease of removal efficiency was found at neutral and basic pH’s, when the salts were added to the solution, signifying inner-sphere surface complexation, largely covalent in character, dominated^47^.

### Desorption and regeneration studies

To minimize the overall operating cost, the usage of spent adsorbent has to be reduced. This can be done by regenerating the adsorbent from the treated effluent. So, desorption tests were conducted, which provides useful information for evaluating the nature of the adsorption process and the regeneration of the spent adsorbent. Usually, these desorption tests are carried using desorbing agents like CaCl_2_, HNO_3_, HCl, EDTA, NaOH, etc., which can desorb the adsorbed ions^48^. In this study, the desorption of diazinon and regeneration of spent pumice was investigated by eluting the diazinon adsorbed pumice with NaOH.

**Figure 7:**
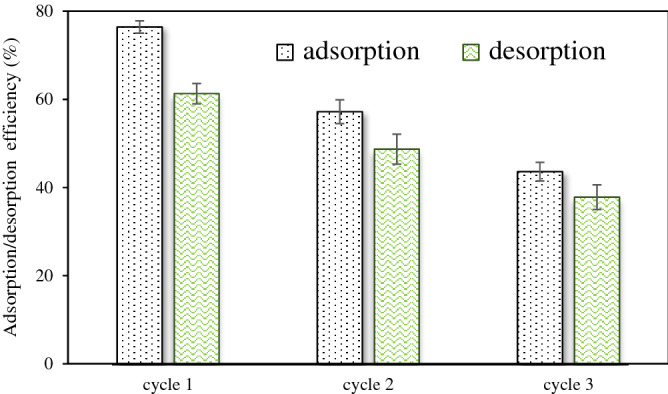
Adsorption–desorption and regeneration studies of pumice.

For desorption tests, 0.1 M NaOH was used on the adsorbed pumice adsorbent. In order to saturate the adsorbent, the first adsorption experiment is conducting by adding 0.75 g of pumice into a 250 ml conical flask containing 5 mg/L of diazinon solution. Then, the flask was placed in a shaker (120 rpm) for 80 minutes to make sure that the adsorbent is completely saturated. The removal percentage of the regenerated pumice was studied in the three cycles of adsorption-desorption processes. The adsorption/desorption study given in Fig. 7 illustrates that adsorption decreased from 76.4 to 43.6%, and desorption reduced from 61.3 to 37.8% after three consecutive cycles. These experimental results indicate that the regenerated pumice adsorbent can be used up to 3 cycles with 43.6 % removal efficiency at the last cycle. So, to minimize the total operation cost (usage of pumice adsorbent), the fresh diazinon polluted water can be treated first with the regenerated adsorbent then get into contact with fresh pumice adsorbent for maximum removal performance.

### Comparison with other adsorbents

In this study, a low-cost and abundant natural adsorbent was used for the diazinon pesticide removal from an aqueous solution, and the performance was found to be excellent. To further validate its performance, the diazinon pesticide removal efficiency was compared with other reported adsorbents. Applications of different adsorbents in the diazinon removal are given in Table 1. As shown in this table, pumice as a natural adsorbent shows a reasonable adsorption capacity for diazinon. Many features like low cost and abundance, availability, and promising adsorption capacity evidence the superiority of pumice over many adsorbents for diazinon removal from aqueous solution.

**Table 1:** Comparison of diazinon adsorption efficiency of different adsorbents.

Adsorbent	Maximum adsorption capacity (mg/g)	References
Ca-montmorillonite	9.5	^39^
FeOH-modified	16.4	
Fe-modified	17.9	
Nanocrystalline MgO	20	^52^
Commercial MgO	12	
Magnetic guar gum	47.17	^50^
Magnetic guar gum-montmorillonite	80	
Acid treated zeolite	15.1	^51^
Cu_2_O nanoparticles modified zeolite	61.73	
Activated coconut shell biochar	9.65	^53^
Phosphoric acid modified coconut shell biochar	10.33	
Chitosan/Carbon nanotube	222.86	^54^
Pumice	20.65	This study

### Isotherms modelling and adsorption kinetics

**Figure 8:**
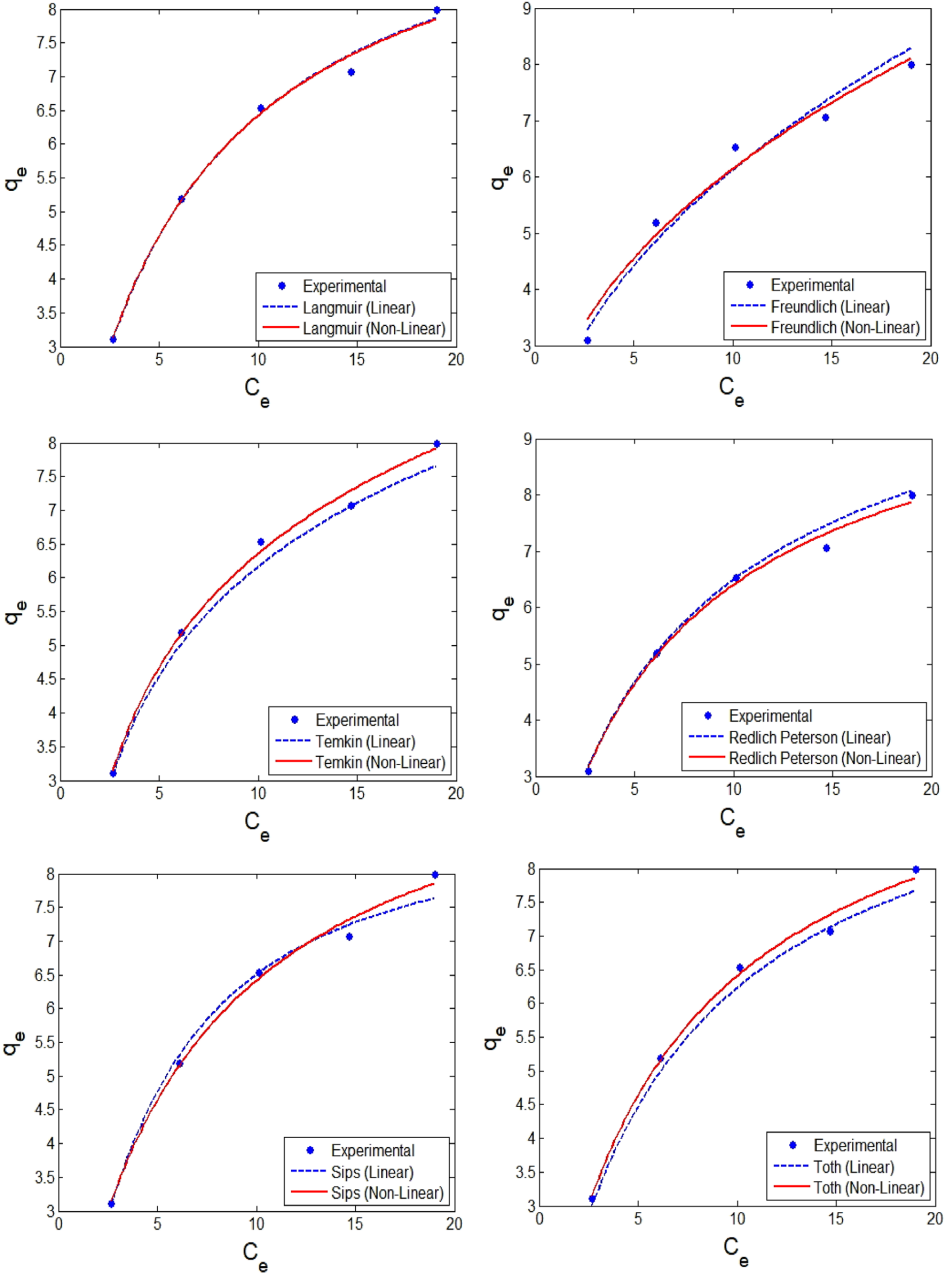
Performance of parameters estimated by linear and non-linear approaches to fit isotherm models that interpret the adsorption of diazinon by pumice (pumice dosage = 1 g/L, and contact time = 30 min, and pH = 3).

The adsorption isotherms describe the relationship between the adsorbent and the amount of analyte present in the solution^49^. In order to better interpret the adsorption of diazinon on pumice, the six well-known isotherm models (2 and 3 parameters models) are evaluated and fitted with the equilibrium adsorption data. As mentioned in “Evaluation of isotherms and kinetic models” section, the model parameters were achieved from both linear and non-linear expressions of adsorption isotherm models. Hence, the isotherm information obtained from the conventional linear method and DEO-based non-linear approach for the equilibrium data for diazinon adsorption onto pumice adsorbent is presented in Fig. 8*.* These results confirm that the model parameters evaluated from non-linear expression better represent the equilibrium isotherms. It is observed that diazinon adsorption onto pumice follows Langmuir and Temkin models, indicating that the monolayer adsorption takes place on the surface, and the adsorption is characterized by a uniform distribution of binding energies. The DEO-based model parameters of Langmuir and Temkin models represent the actual adsorption process and hence resulted in a higher R^2^ of 0.995 and 0.994, respectively. These outcomes are very similar to the results reported by Nikzad et al.^50^, who studied the diazinon adsorption using magnetic guar gum-montmorillonite.

Even though the R^2^ value is the metric that is used by most researchers to validate the model predictions, but this metric cannot be representing the true error residuals. Hence, this cannot be the only metric to appraise the performance. Sometimes, researchers calculate another statistical metric like RMSE. This metric definitely provides good information on the overall error residuals but does not give enough information on the error distribution like overfitted or underfitting. Therefore, other important metrics like SAE, SSE and ARE should be determined. These metrics for the isotherm models that were employed to represent the diazinon adsorption onto pumice adsorbent are evaluated and shown in Table 2. It can be observed that the RMSE values for Langmuir and Temkin models are the lowest for optimized non-linear model parameters, thus further confirming that the non-linear model captures the inherent mechanisms. The lower values of other metrics also confirm the same. These results depict that a monolayer adsorption phenomenon occurs, and uniform distribution of binding energies at the adsorbent surface occurs during the adsorption of diazinon by pumice from an aqueous solution.

**Table 2:** Isotherm models for adsorption of diazinon onto pumice*.

Models →	Freundlich		Langmuir		Temkin		Redlich–Peterson		Sips		Toth	
Statistical metrics ↓	L	NL	L	NL	L	NL	L	NL	L	NL	L	NL
Parameters →	q_e_: 20.65 b_F_: 0.472	K_F_: 20.27 b_F_: 0.433	K_L_: 10.48 b_L_: 0.159	K_L_: 10.42 b_L_: 0.161	B_T_: 1.412 K_T_: 2.33	B_T_: 1.370 K_T_: 2.43	K_R_: 1.813 a_R_: 0.220 α: 0.923	K_R_: 1.763 a_R_: 0.191 α: 0.964	K_s_: 8.923 b_s_: 0.1592 n_s_: 0.814	K_s_: 10.54 b_s_: 0.162 n_s_: 0.962	K_th_: 10.35 b_th_: 0.159 n_th_: 0.986	K_th_: 10.73 b_th_: 0.180 n_th_: 0.947
R^2^	0.972	0.981	0.993	0.995	0.989	0.994	0.982	0.989	0.978	0.986	0.983	0.989
SAE	1.474	1.258	0.533	0.529	0.882	0.540	0.580	0.584	0.661	0.564	0.989	0.573
SSE	0.458	0.366	0.097	0.095	0.264	0.084	0.104	0.094	0.178	0.096	0.247	0.096
ARE	12.883	12.762	3.917	3.882	6.769	4.342	4.629	4.664	4.725	4.343	6.346	4.489
RMSE	0.338	0.282	0.156	0.149	0.257	0.145	0.161	0.154	0.211	0.155	0.248	0.155

*Linear (L); non-linear (NL).

$q_{e,exp}^{i} ,q_{e, pred}^{i}$ are q_e_ values of experimental and predicted values respectively. p is the number of parameters; n is the number of experimental runs.

Units: Langmuir: K_L_ (mg/g). b_L_ (L/mg). Freundlich: K_F_ (mg^1-(1/n)^ L^(1/n)^ g^-1^) and b_F_ is dimensionless. Temkin: K_T_ (L/mg). b_T_ (J/mol). Redlich – Peterson: K_R_ (L/g) and a_R_(L/mg) ^nP^ and n_R_ is dimensionless. Sips: K_S_,(mg/g). b_s_ (L/mg)^n^ and n_s_ is dimensionless. Toth: K_th_ (L/g). b_th_ (mg/L)^n^ and n_th_ is dimensionless exponent.

**Figure 9:**
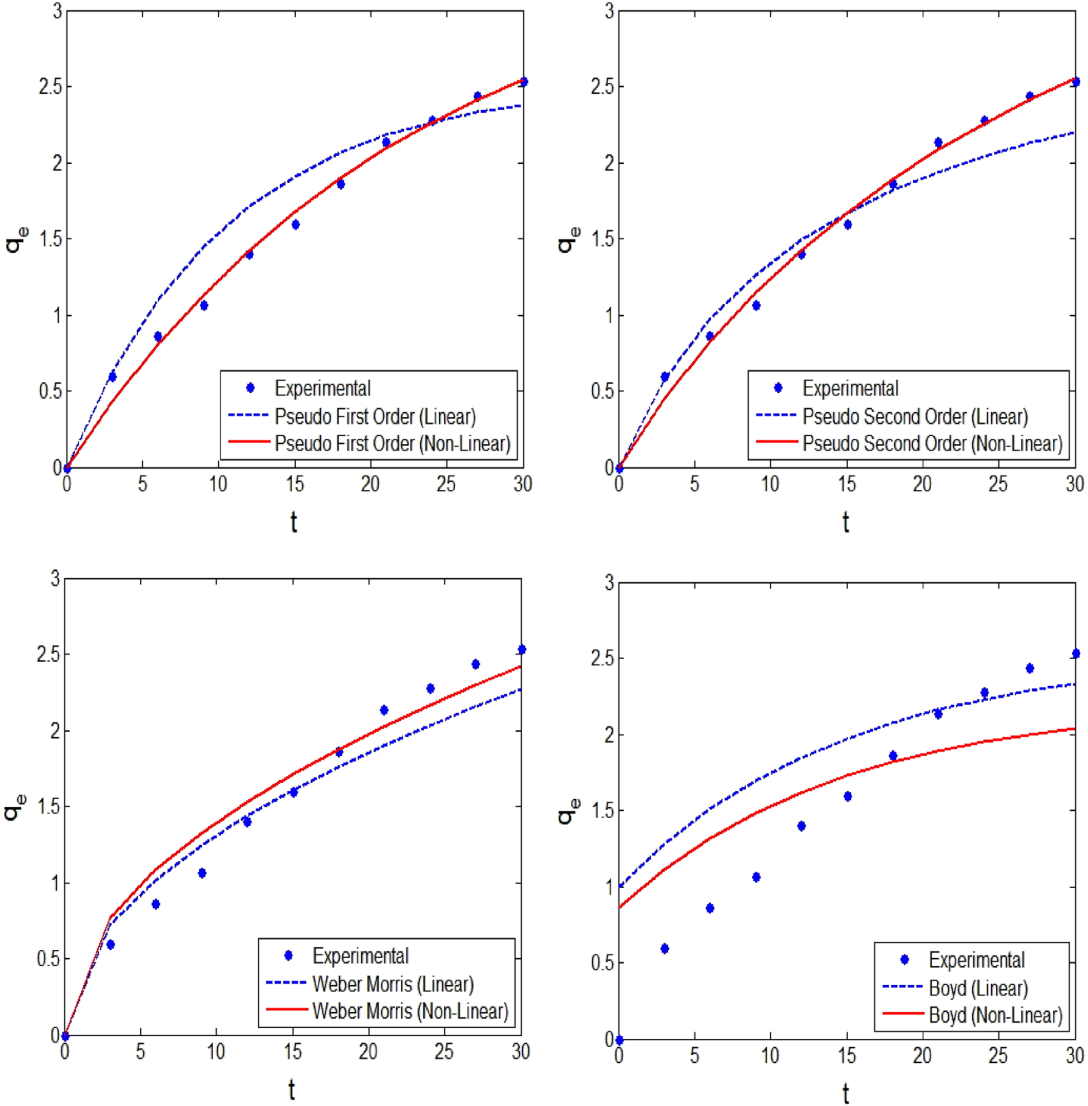
Kinetic plots for the adsorption of diazinon by pumice (initial diazinon concentration = 50 mg/L, pumice dosage = 1 g/L, and pH = 3).

The adsorption rate of the adsorbent in the solution for diazinon can be understood by investigating the kinetic behaviour of the adsorption process. The adsorption kinetics were analyzed using the conventional and inter-molecular diffusion kinetic models. The experiments are conducted in the range of 3–30 min. These kinetic models are generally non-linear expressions, but most researchers linearize these models to evaluate the respective model parameters using the least square regression fit. As mentioned in recent literature^29^, the model parameters evaluated will under/overestimate and hence misinterpret the mechanisms in the adsorption process. Therefore, in this study, DEO is implemented to evaluate the model parameters from the non-linear kinetic models. The performance “of these optimized model parameters (non-linear model) against the conventional least square fit is shown in Fig. 9. It is evident that DEO-based model parameters better represent the kinetic behaviour and hence can be used to interpret the adsorption rate and kinetics involved in the diazinon adsorption onto pumice. From the result presented in Fig. 9, it is seen that the diazinon adsorption onto pumice follows pseudo 1^st^ and 2^nd^ order kinetics, which resulted in *R*^2^ > 0.99. These outcomes are very similar to the reported results for diazinon removal from aqueous solutions^50,51^.

The important statistical metrics that appraise the significance of linear and non-linear approaches for interpreting kinetics using various kinetic models are presented in Table 3. It is seen that the DEO-based kinetic parameters better capture the inherent kinetic behaviour in diazinon adsorption onto pumice, and hence, resulted in higher *R*^2^ and lower ARE, SSE, SAE, and RMSE. Therefore, these statistical metrics further confirm the praiseworthy and creditability of the DEO approach.

**Table 3:** Kinetic models for diazinon removal by pumice**.

Models →	PFO		PSO		Weber—Morris		Boyd	
Statistical metrics ↓	L	NL	L	NL	L	NL	L	NL
Parameters →	K_1_: 0.0012 q_e_: 84.32	K_1_: 0.044 q_e_: 3.49	K_2_: 0.022 q_e_: 3.233	K_2_: 0.0054 q_e_: 5.4342	K_id_:0.415	K_id_:0.442	B: 0.0675 q_e_: 2.533	B: 0.068 q_e_: 2.210
R^2^	0.9368	0.9910	0.9623	0.9914	0.9796	0.9852	0.9515	0.9709
SAE	64.835	27.126	47.137	25.581	53.080	51.808	164.801	133.266
SSE	0.460	0.048	0.371	0.044	0.346	0.229	1.711	1.320
ARE	1.758	0.524	1.622	0.514	1.622	1.372	3.410	3.292
RMSE	0.339	0.109	0.305	0.105	0.294	0.239	0.654	0.574

**Linear (L). Non-linear (NL). Pseudo first order (PFO) and Pseudo second-order (PSO).

$q_{e,exp}^{i} ,q_{e,pred}^{i}$ are the q_e_ values of the experimental and predicted values, respectively.

*p* is the number of parameters. *n* is the number of experimental runs.

Pseudo-first-order model (PFO) & pseudo-second-order model (PSO).

Units: q_e_ (mg/g), K_1_ (min^−1^), K_2_ (g mg^−1^ min^-1^), K_id_ (mg/g min^0.5^), Di (cm^2^).

### Thermodynamic studies of diazinon pesticide adsorption using pumice adsorption

In this work, thermodynamic studies were performed at the optimal conditions of the process variables and temperatures of 293, 303, 318, and 323 K. The thermodynamic equilibrium constant (*Ke*°) is calculated from the Eq. (a.3). From the isotherm studies, it was observed that diazinon onto pumice follows the Langmuir isotherm model; hence the K_L_ is the Langmuir equilibrium constant. After that, ${\text{K}}_{{\text{L}}}^{{\text{m}}}$ (L/mol) is calculated by multiplying with a molecular weight of adsorbate and translating it into appropriate units. This value is multiplied with the unitary standard concentration of the adsorbate (1 mol/L) and dividing with the activity coefficient (γ). Since the adsorbate solution in this study is much diluted, hence the activity coefficient can be assumed as unity. The important parameters like standard enthalpy (ΔH°), Gibbs free energy (ΔG°) and standard entropy (ΔS°) are obtained from the thermodynamic relations given in Eq. (a.1) & Eq. (a.2) are presented in Table S7. The computed values of ΔG° were negative, showing a natural adsorption process, and it is decreasing with the increase of temperature. Thus, higher temperatures supported the diazinon adsorption onto pumice due to a greater force of adsorption. The positiveness of ΔH° depicts that the endothermic nature of the adsorption process. Also, the positive values of ΔS° indicate the increase in randomness at the solid–liquid interface during the adsorption process, as well as a greater affinity between diazinon and adsorbent.

## Conclusions

A low-cost and natural pumice adsorbent was used to remove organophosphorus diazinon pesticide from water bodies. The characterization of pumice adsorbent before and after the adsorption process is investigated by analytical instruments. To understand the influence of independent process variables like pH, initial diazinon concentration, pumice adsorbent, and contact time on the diazinon pesticide removal, a systematic statistical approach is used, and the experimental design matrix is developed. The RSM-CCD framework modeled diazinon removal, and the interactive effects of process variables on the removal percentage are examined. To predict the diazinon removal (%) at different process conditions, a quadratic model is developed using ANOVA in RSM. The model validation indicated that this quadratic model is well-fitted model with R^2^ = 0.9997, p-value < 0.001, and Lack of fit = 0.872. ANN approach was used to develop a data-driven model. This approach suggested a topology of 4:8:1 with backpropagation ANN, whose model predictions resulted in R^2^ = 0.9991. To identify the optimum process variables that result in the highest removal efficiency, GA and RSM techniques are used. RSM based optimization results showed the maximum diazinon removal efficiency of 76.3% is obtained at initial diazinon concentration = 6.288 mg/L, pH = 3, pumice dosage = 4 g/L, and contact time = 30 min. The GA optimization results were very similar to the RSM technique. The pumice showed a promising result of regeneration. The ionic strength results showed that the removal efficiency is suppressed significantly at acidic pH, whereas it exhibits no significant decrease at neutral and basic pH’s. Diazinon adsorption isotherms were also investigated, and the experimental data follows Langmuir isotherm. It was found that the diazinon adsorption could be well described by the pseudo-first and second-order kinetic models. Diazinon removal by pumice showed the favourable, spontaneous endothermic nature of the adsorption process. Therefore, pumice can be considered as a potential efficient adsorbent for the removal of pesticides from aqueous solutions.

## Supplementary Information

Supplementary Information.
