# Supplementary material for: Process optimization and enhancement of pesticide adsorption by porous adsorbents by regression analysis and parametric modelling
Source: Sci Rep. 2021 Jun 3;11:11719. doi: 10.1038/s41598-021-91178-3 (PMC8175395; doi:10.1038/s41598-021-91178-3)
Supplement: Supplementary file 1 — Supplementary Information. [file 41598_2021_91178_MOESM1_ESM.docx]

***Electronic Supplementary material (ESM)***

**Process optimization and enhancement of pesticide adsorption by porous adsorbents by regression analysis and parametric modelling**

Mohammad Hadi Dehghani ^a,b*^, Amir Hessam Hassani^c^, Rama Rao Karri ^d^, Bahareh Younesi^c**^ Mansoureh Shayeghi ^e^, Mehdi Salari ^f^ , Ahmad Zarei ^g^ , Mahmood Yousefi ^a^, Zoha Heidarinejad ^h^

*^a^ Department of Environmental Health Engineering, School of Public Health, Tehran University of Medical Sciences, Tehran, Iran*

*^b^ Institute for Environmental research, Center for Solid Waste Research, Tehran University of Medical Sciences, Tehran, Iran*

*^c^Department of Environmental Engineering, Faculty of Environment and Energy, Science and Research Branch, Islamic Azad University, Tehran, Iran*

*^d^ Petroleum and Chemical Engineering, Faculty of Engineering, Universiti Teknologi Brunei, Brunei Darussalam*

*^e^ Department of Medical Entomology and Vector Control, School of Public Health, Tehran University of Medical Sciences, Tehran, Iran*

*^f^ Student Research Committee, Department of Environmental Health Engineering, School of Public Health, Hamadan University of Medical Sciences, Hamadan, Iran*

*^g^ Department of Environmental Health Engineering, School of Health, Social Determinants of Health Research Center, Gonabad University of Medical Sciences, Gonabad, Iran*

*^h^ Food Health Research Center, Hormozgan University of Medical Sciences, Bandar Abbas, Iran and Department of Environmental Health Engineering, Faculty of Health, Hormozgan University of Medical Sciences, Bandar Abbas, Iran*

**^*^Corresponding author:** Department of Environmental Health Engineering, School of Public Health, Tehran University of Medical Sciences, Tehran, Iran; email: hdehghani@tums.ac.ir (M.H. Dehghani).

**^**^Corresponding author:** Department of Environmental Engineering, Faculty of Environment and Energy, Science and Research Branch, Islamic Azad University, Tehran, Iran; email: b_unesei@yahoo.com (B. Younesi).

**Table S1-S7**

Table S1. Process parameters and the levels of each parameter

| Variable | Symbol | -α | -1 | 0 | +1 | +α |
| --- | --- | --- | --- | --- | --- | --- |
| pH | X_1_ | 3 | 4.5 | 6 | 7.5 | 9 |
| Contact time (min) | X_2_ | 5 | 11.25 | 17.5 | 23.75 | 30 |
| Pumice dosage (g/L) | X_3_ | 1 | 1.75 | 2.5 | 3.25 | 4 |
| Initial diazinon concentration (mg/L) | X_4_ | 1 | 13.25 | 25.5 | 37.75 | 50 |

Table S2 . Experimental design matrix for diazinon adsorption by pumice adsorbent

| Run No. | pH | Time (min) | adsorbent dose (g/L) | Pollutant concentration (mg/L) | Actual response | Predicted response | |
| --- | --- | --- | --- | --- | --- | --- | --- |
|  |  |  |  |  |  | ANN | CCD-RSM |
| 1 | 4.5 | 23.75 | 1.75 | 13.25 | 53.30 | 53.30 | 53.29 |
| 2 | 4.5 | 23.75 | 1.75 | 37.75 | 29.50 | 29.50 | 29.69 |
| 3 | 4.5 | 11.25 | 3.25 | 13.25 | 49.20 | 49.20 | 49.24 |
| 4 | 6 | 17.5 | 2.5 | 1 | 48.90 | 48.90 | 48.46 |
| 5 | 7.5 | 23.75 | 3.25 | 37.75 | 28.50 | 28.50 | 28.49 |
| 6 | 4.5 | 23.75 | 3.25 | 13.25 | 61.40 | 61.40 | 61.62 |
| 7 | 7.5 | 11.25 | 3.25 | 37.75 | 18.30 | 18.30 | 18.30 |
| 8 | 6 | 17.5 | 2.5 | 50 | 1.30 | 1.30 | 1.56 |
| 9 | 9 | 17.5 | 2.5 | 25.5 | 31.60 | 29.46 | 31.43 |
| 10 | 4.5 | 11.25 | 3.25 | 37.75 | 26.20 | 26.20 | 26.34 |
| 11 | 6 | 30 | 2.5 | 25.5 | 48.50 | 54.07 | 48.34 |
| 12 | 7.5 | 23.75 | 1.75 | 37.75 | 18.50 | 18.50 | 18.45 |
| 13 | 4.5 | 23.75 | 3.25 | 37.75 | 38.80 | 35.51 | 38.57 |
| 14 | 6 | 17.5 | 4 | 25.5 | 46.10 | 46.10 | 45.94 |
| 15 | 6 | 17.5 | 2.5 | 25.5 | 39.10 | 39.30 | 39.43 |
| 16 | 6 | 17.5 | 2.5 | 25.5 | 38.90 | 39.30 | 39.43 |
| 17 | 4.5 | 11.25 | 1.75 | 13.25 | 41.50 | 41.50 | 41.70 |
| 18 | 6 | 17.5 | 2.5 | 25.5 | 39.60 | 39.30 | 39.43 |
| 19 | 7.5 | 11.25 | 1.75 | 37.75 | 9.10 | 7.23 | 9.07 |
| 20 | 6 | 17.5 | 1 | 25.5 | 28.40 | 34.58 | 28.38 |
| 21 | 3 | 17.5 | 2.5 | 25.5 | 50.30 | 50.30 | 50.29 |
| 22 | 6 | 5 | 2.5 | 25.5 | 26.60 | 26.60 | 26.58 |
| 23 | 7.5 | 11.25 | 3.25 | 13.25 | 41.60 | 41.60 | 41.60 |
| 24 | 6 | 17.5 | 2.5 | 25.5 | 39.90 | 39.30 | 39.43 |
| 25 | 7.5 | 11.25 | 1.75 | 13.25 | 32.70 | 32.70 | 32.92 |
| 26 | 6 | 17.5 | 2.5 | 25.5 | 39.40 | 39.30 | 39.43 |
| 27 | 7.5 | 23.75 | 3.25 | 13.25 | 51.60 | 52.89 | 51.94 |
| 28 | 4.5 | 11.25 | 1.75 | 37.75 | 18.60 | 17.70 | 18.25 |
| 29 | 7.5 | 23.75 | 1.75 | 13.25 | 42.40 | 42.40 | 42.45 |
| 30 | 6 | 17.5 | 2.5 | 25.5 | 39.70 | 39.30 | 39.43 |

Table S3. Various forms of isotherm models

| **Type of Isotherm** | **Non-linear expression** | **Linear expression** | **Plot (linear)** | **Parameters** |
| --- | --- | --- | --- | --- |
| ***Two parameters isotherm models*** | | |  |  |
| Freundlich model | $q_{e}=K_{F}C_{e}^{1/n}$ | $\ln\left( q_{e} \right)=\ln\left( K_{F} \right)+\frac{1}{n}\ln\left( C_{e} \right)$ | *ln (q_e_) vs ln(C_e_)* | *K_F_ and n* |
| Langmuir (Type 1) | $q_{e}=\left( \frac{K_{L}bC_{e}}{1+bC_{e}} \right)$ | $\frac{1}{q_{e}}=\frac{1}{K_{L}}+\left( \frac{1}{K_{L}b} \right)\frac{1}{C_{e}}$ | *1/q_e_ vs 1/C_e_* | *K_L_ and b* |
| Langmuir (Type 2) |  | $\frac{C_{e}}{q_{e}}=\frac{1}{K_{L}}C_{e}+\frac{1}{K_{L}b}$ | *C_e_/q_e_ vs. C_e_* | *K_L_ and b* |
| Langmuir (Type 3) |  | $q_{e}=K_{L}-\frac{1}{b}\frac{q_{e}}{C_{e}}$ | *q_e_ vs. q_e_/C_e_* | *K_L_ and b* |
| Langmuir (Type 4) |  | $\frac{q_{e}}{C_{e}}=K_{L}b-bq_{e}$ | *q_e_/C_e_ vs. q_e_* | *K_L_ and b* |
| Temkin | $C_{e}=\frac{1}{K_{T}}\exp\left(\frac{b_{T}q_{e}}{RT}\right)$ | $q_{e}=B_{T}\left(\ln(K_{T})+\ln C_{e}\right)$ | *q_e_ vs ln C_e_* | *K_T_ and B_T_* |
| ***Three parameters isotherm models*** | | | | |
| Redlich - Peterson | $q_{e}=\left( \frac{K_{R}C_{e}}{1+a_{R}C_{e}^{\alpha}} \right)$ | $\log\left( K_{R}\frac{C_{e}}{q_{e}}-1 \right)$ $=\log\left( a_{R} \right)+\alpha\log\left( C_{e} \right)$ | Thru trial and approximation method | *K_R_, a_R,_ and α* |
| Sips | $q_{e}=\left( \frac{K_{S}b_{S}{C_{e}}^{1/n_{S}}}{1+b_{S}{C_{e}}^{1/n_{S}}} \right)$ | $\frac{1}{q_{e}}=\frac{1}{K_{s}}+\left( \frac{1}{K_{s}b_{S}} \right)\frac{1}{{C_{e}}^{1/n_{S}}}$ | Thru trial and approximation method | *K_S_ , b_S_, and n_S_* |
| Toth | $q_{e}=\frac{K_{th}C_{e}}{\left( b_{th}+{C_{e}}^{n} \right)^{1/n}}$ | $\frac{1}{{q_{e}}^{n}}=\frac{1}{K_{th}}+\left( \frac{b_{th}}{K_{th}} \right)\frac{1}{{C_{e}}^{n}}$ | Thru trial and approximation method | *K_th_, b_th_ and n_th_* |

**Table S4***.* Summary of different conventional type of kinetics both in linear and non-linear forms generally used to estimate the adsorption rate along with corresponding plot to estimate the parameters

| **Type of kinetics** | **Non-linear form** | **Linear form** | **Plot** | **Parameters** |
| --- | --- | --- | --- | --- |
| *Pseudo 1^st^ order* | $q=q_{e}\left( 1-e^{-k_{1}t} \right)$ | $\ln(1-\frac{q}{q_{e}})=-k_{1}t$ | *ln(1-q/q_e_) vs. t* | k_ad_ = -slope |
| *Pseudo 2^nd^ order (Type 1)* | $q=\left( \frac{K_{2}q_{e}^{2}}{1+tK_{2}q_{e}} \right)t$ | $\frac{t}{q}=\frac{1}{k_{2,1}{q_{e}}^{2}}+\frac{1}{q_{e}}t$ | *t/q vs. t* | q_e_ = 1/slope, k_2,1_=slope^2^/intercept |
| *Pseudo 2^nd^ order (Type 2)* |  | $\frac{1}{t}=k_{2,2}q_{e}^{2}\left( \frac{1}{q} \right)-k_{2,2}q_{e}$ | *1/t vs.1/q* | q_e_ = -slope/intercept, k_2,2_= intercept^2^/slope |
| *Pseudo 2^nd^ order (Type 3)* |  | $\frac{1}{q}=\left( \frac{1}{k_{2,3}q_{e}^{2}} \right)\frac{1}{t}+\frac{1}{q_{e}}$ | *1/q vs.1/t* | q_e_ = 1/intercept,  k_2,3_= intercept^2^/slope |
| *Pseudo 2^nd^ order (Type 4)* |  | $\frac{1}{q_{e}-q}=\frac{1}{q_{e}}+k_{2,4}t$ | *1/(q_e_-q) vs. t* | q_e_ = 1/intercept, k_2,4_=slope |
| *Pseudo 2^nd^ order (Type 5)* |  | $\frac{q}{t}=-k_{2,5}q_{e}*q+k_{2,5}q_{e}^{2}$ | *q/t vs. q* | q_e_ = - intercept/slope, k_2,5_=slope^2^/intercept |
| *Pseudo 2^nd^ order (Type 6)* |  | $q=q_{e}-\left( \frac{1}{k_{2,6}q_{e}} \right)\frac{q}{t}$ | *q vs. q/t* | q_e_ = intercept,  k_2,6_=-1/slope*intercept |
| *Weber - Morris model* | $q=k_{id}\sqrt{t}$ |  | *log(q) vs. 0.5log(t)* | k_id_ = exp (intercept) |
| *Boyd model* | $\frac{q}{q_{e}}=1-\frac{6}{\pi^{2}}\exp(-B_{t})$ | $B_{t}=-0.4977-\ln(1-F)$  for *F* (= *q/q_e_*) > 0.85  $B=\frac{\pi^{2}D_{i}}{r^{2}}$ | *B_t_ vs t* | *D_i_* |

**Table S5.** ANOVA for the quadratic model to predict diazinon adsorption onto pumice

| Source | df | Mean square | F-value | | p-value |
| --- | --- | --- | --- | --- | --- |
| Model | 14 | 385.17 | 3741.56 | | <0.001 |
| BPA concentration | 1 | 3299.42 | 32050.44 | | <0.001 |
| Adsorbent dose | 1 | 462.88 | 4496.42 | | <0.001 |
| Time | 1 | 710.68 | 6903.55 | | <0.001 |
| pH | 1 | 533.93 | 5186.55 | | <0.001 |
| AB | 1 | 4.20 | 40.82 | | <0.001 |
| AC | 1 | 1.32 | 12.85 | | <0.05 |
| AD | 1 | 0.16 | 1.55 | | 0.232 |
| BC | 1 | 0.64 | 6.22 | | <0.05 |
| BD | 1 | 0.02 | 0.22 | | 0.647 |
| CD | 1 | 0.30 | 2.94 | | 0.107 |
| AÂ² | 1 | 3.48 | 33.82 | | <0.001 |
| BÂ² | 1 | 6.69 | 64.96 | | <0.001 |
| CÂ² | 1 | 8.87 | 86.19 | | <0.001 |
| DÂ² | 1 | 356.71 | 3465.07 | | <0.001 |
| Residual | 15 | 0.10 | - | | - |
| Lack of Fit | 10 | 0.08 | 0.58 | | 0.782 |
| R^2^ | | Adjusted R^2^ | | Predicated R^2^ | |
| 0.9997 | | 0.9994 | | 0.9989 | |

**Table S6.** Model validation at optimal conditions

| Initial diazinon concentration (mg/L) | Pumice dosage (g/L) | pH | Contact time (min) | Removal efficiency (%) | |
| --- | --- | --- | --- | --- | --- |
| 6.28 | 4 | 3 | 30 | 76.3 | RSM |
| 6.266 | 4 | 3 | 30 | 76.5 | GA |
| 6.28 | 4 | 3 | 30 | 77.0 (±1.7) | Expt |

**Table S7.** Thermodynamic parameters for the removal of diazinon by pumice

| Temperature (K) | K_L_ (L/mg) | K_e_^o^ | ln (K_e_^o^) | ΔG^o^  (kJ/mol) | ΔH° (kJ/mol) | ΔS°  (kJ/mol-K) |
| --- | --- | --- | --- | --- | --- | --- |
| 293.00 | 10.42 | 4.0E+05 | 12.89 | -31.40 | 86.68 | -5.97 |
| 303.00 | 9.23 | 3.5E+05 | 12.77 | -32.16 |  |  |
| 318.00 | 8.56 | 3.3E+05 | 12.69 | -33.56 |  |  |
| 323.00 | 7.95 | 3.0E+05 | 12.62 | -33.89 |  |  |

**Figs S1-S8**


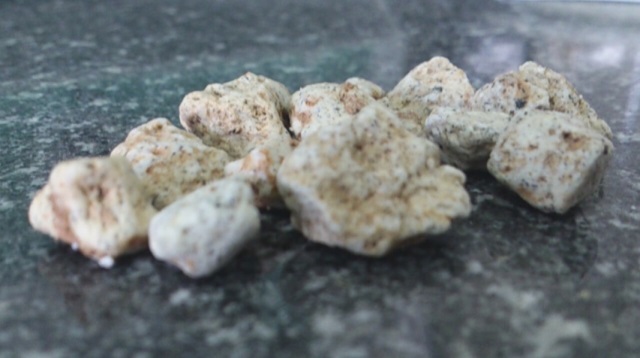


**Fig. S1.** Natural pumice stone used in this study

^
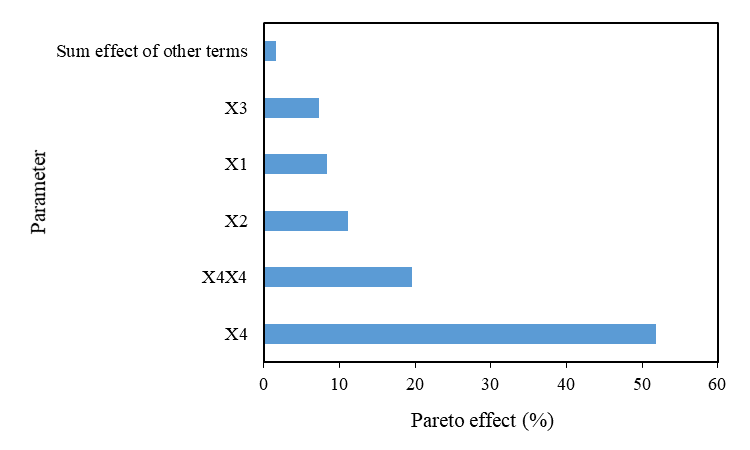
^

**Fig. S2.** Pareto chart for the effect of each term on the response


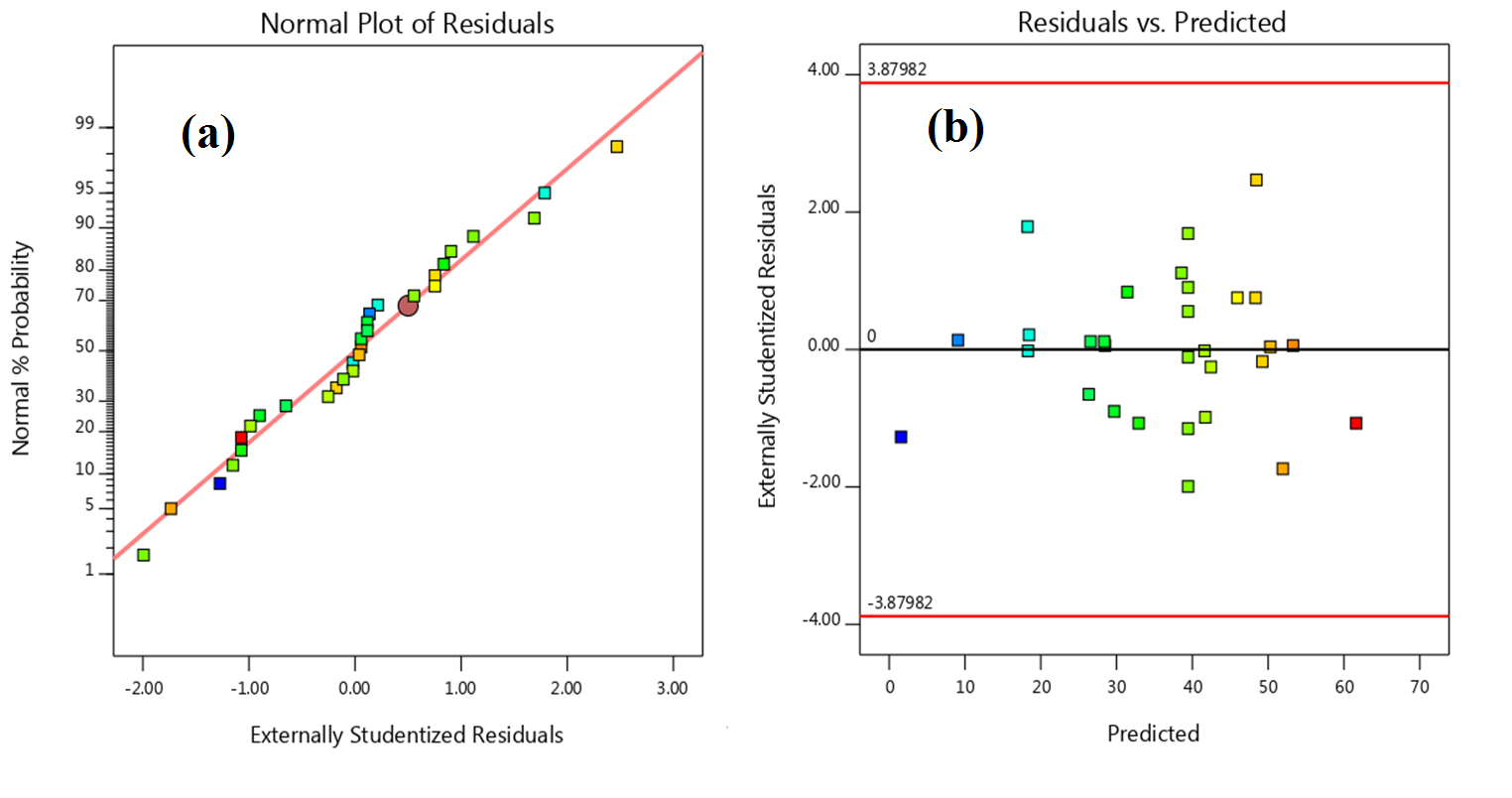


**Fig. S3.** Adequacy plots of the quadratic model


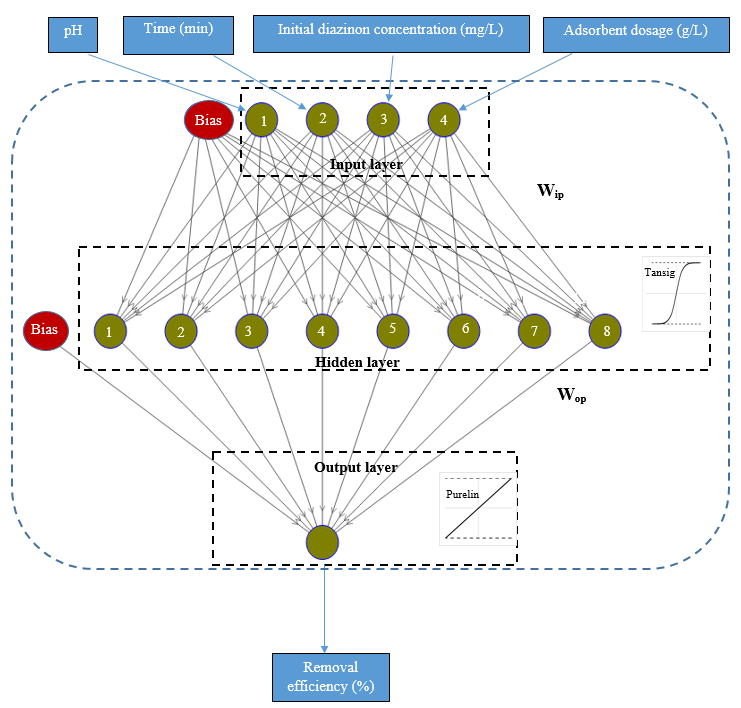


**Fig. S4.** Neural network architecture with 4-8-1 topology

***
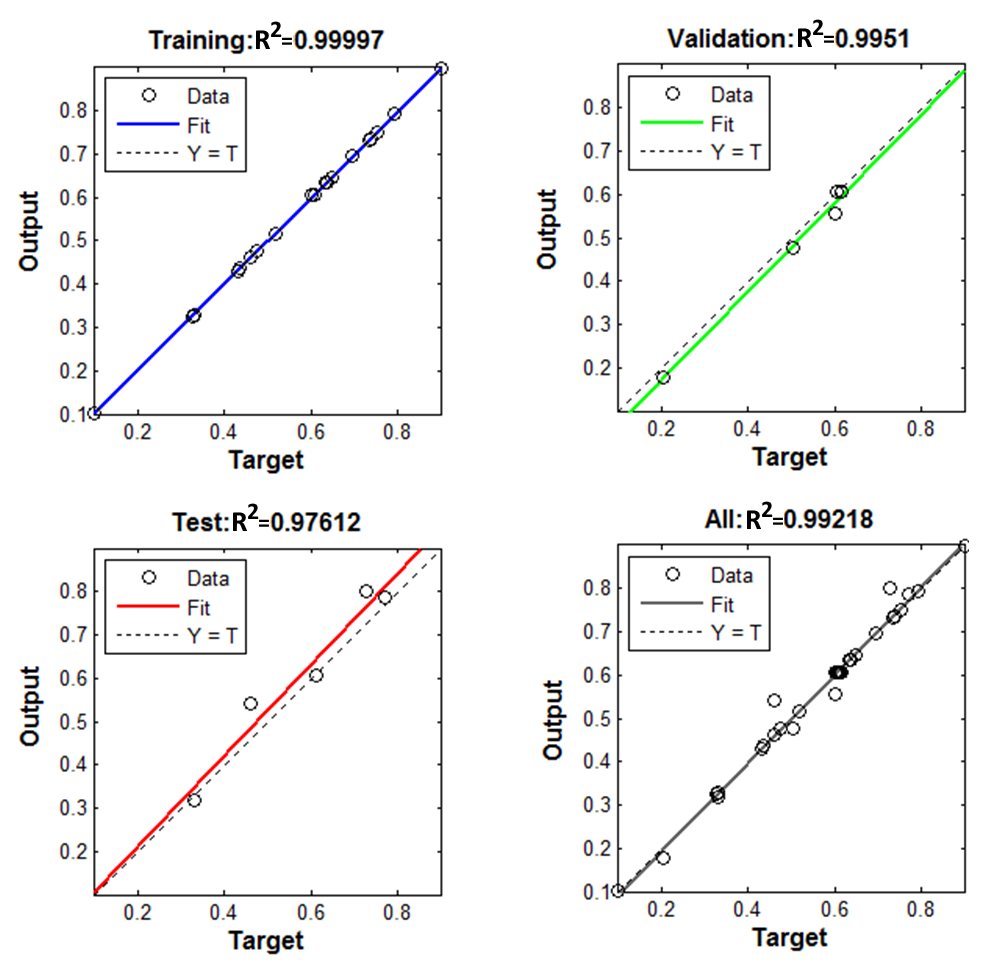
***

**Fig. S5.** The performance of the ANN model with 4-8-1 topology


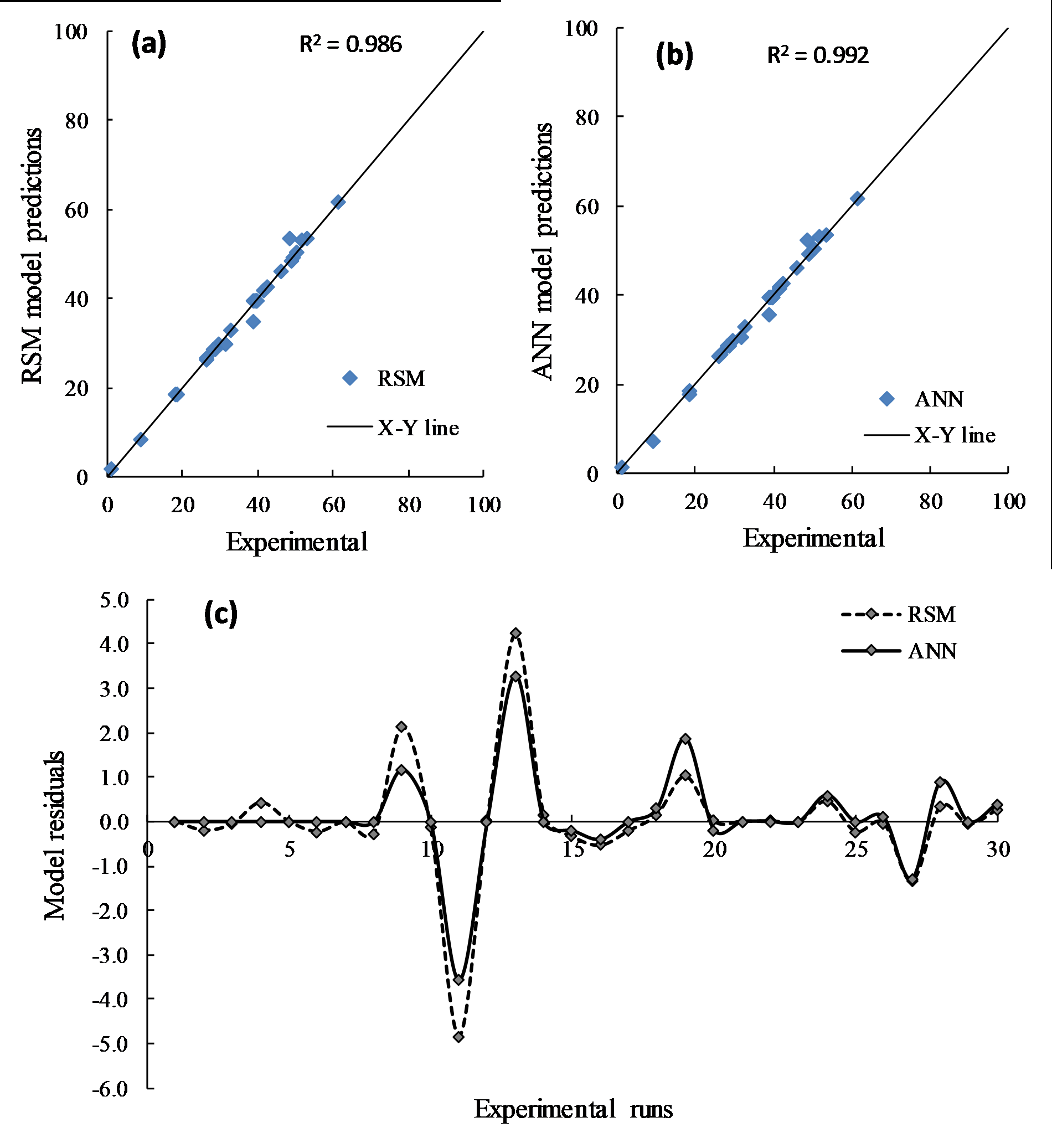


**Fig. S6.** Performance of RSM and ANN model predictions


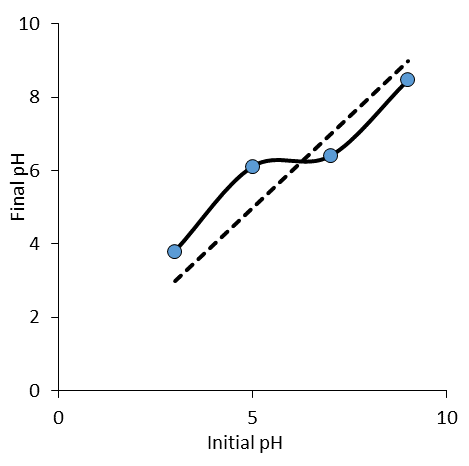


**Fig. S7.** pH_ZPC_ for pumice adsorbent

**
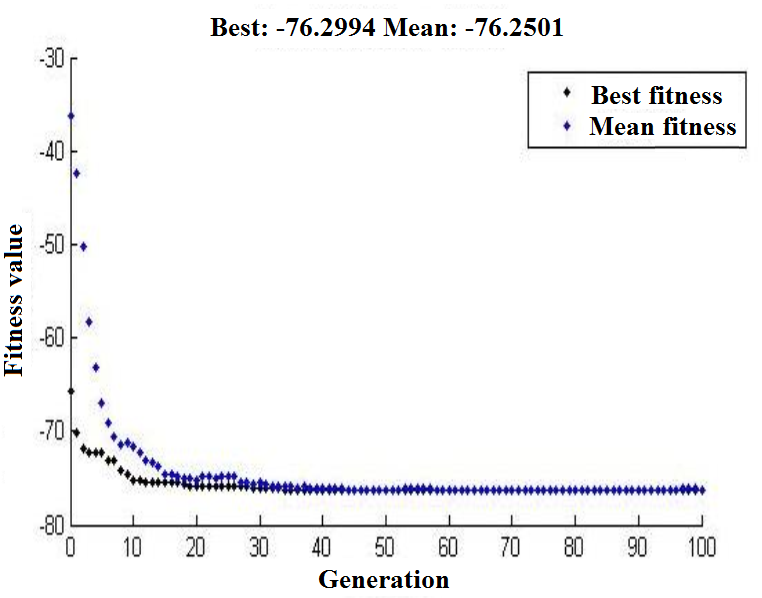

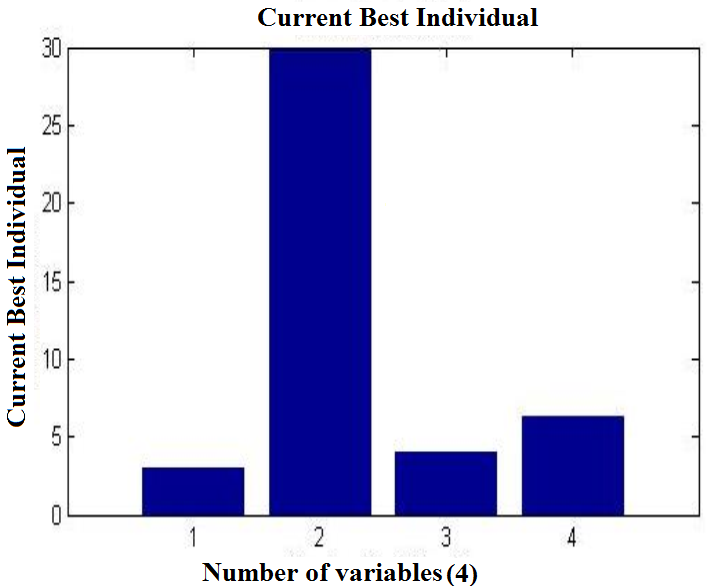
**

**
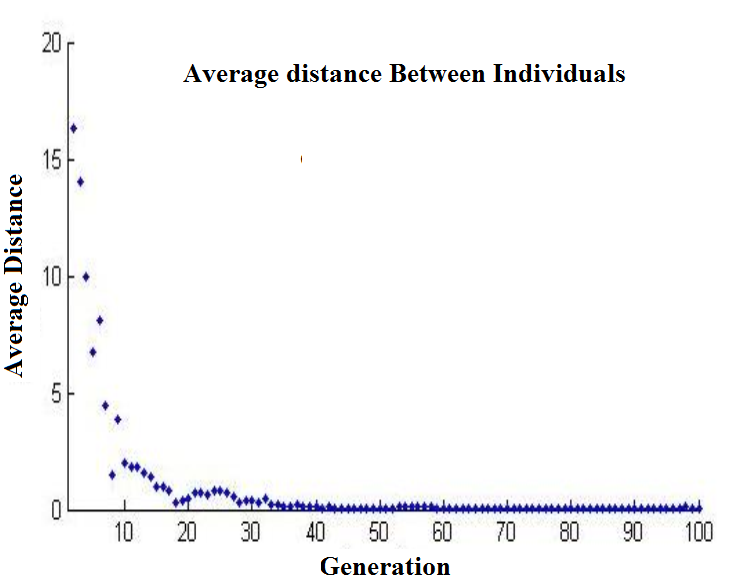

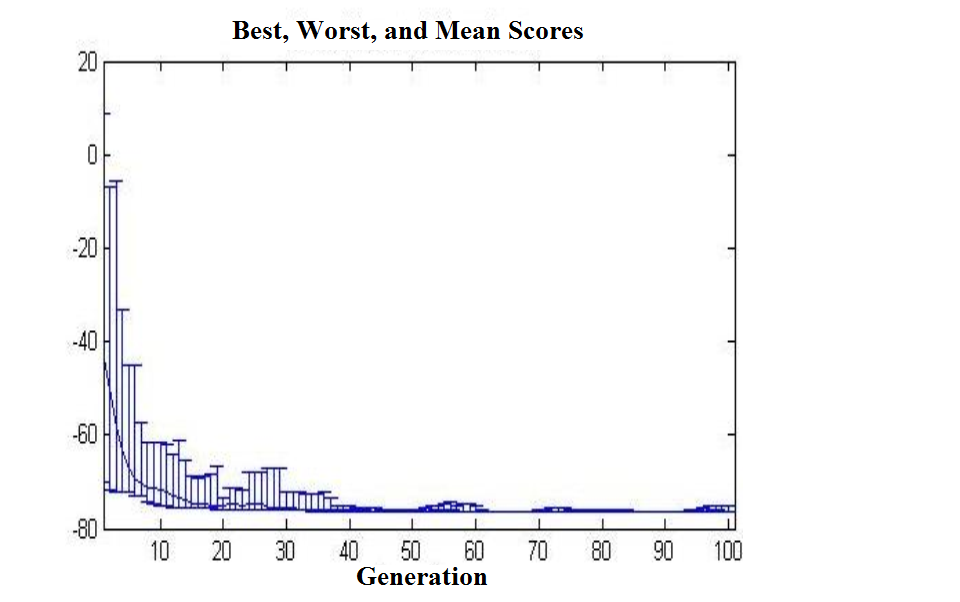
**

**Fig. S8.** Plots of significant statistical factors in the implementation of the GA technique
